# COVID-19 pandemic: a multinational report providing professional experiences in the management of mental health of elderly

**DOI:** 10.1017/S1041610220001027

**Published:** 2020-05-29

**Authors:** Renato Sobral Monteiro-Junior, Lara S. F. Carneiro, Maria Lage Barca, Kari Midtbø Kristiansen, Cristina Andrade Sampaio, Desirée Sant’Ana Haikal, Lêda Antunes, Luana Lemos Leão, Andrea Camaz Deslandes

**Affiliations:** 1 Post-Graduate Program of Health Sciences, Montes Claros State University, Montes Claros, Minas Gerais, Brazil; 2 Aroldo Tourinho Hospital, Montes Claros, Minas Gerais, Brazil; 3 Higher Institute of Educational Sciences (ISCE, Douro), Penafiel, Portugal; 4 University Institute of Maia (ISMAI), Maia, Portugal; 5 Research Centre in Sports Sciences, Health Sciences and Human Development, CIDESD, GERON Research Community, Vila Real, Portugal; 6 Norwegian Advisory Unit for Ageing and Health, Vestfold Hospital Trust, Tønsberg, Norway; 7 Institute of Psychiatry, Federal University of Rio de Janeiro, Rio de Janeiro, Brazil

## Introduction

There is a huge amount of information on SARS-CoV-2 (COVID-19), but its influence on mental health is still lacking. Although all age groups are at risk of contracting COVID-19, older people face significant risk of developing severe illness (Kluge, 2020). The old age is an independent factor for the COVID-19-related death (RR = 9.45) (Zhao *et al.*, 2020). This pandemic might affect this population’s mental health. Retrospective studies of the Severe Acute Respiratory Syndrome epidemic demonstrated that suicide rates raised among elderly during this period (Kluge, 2020). This data emphasize the urgency of studying COVID-19 mental health impact in real time, since it has brought consequences such as psychological suffering, fear, depression and anxiety (Courtin and Knapp, 2017; Fiorillo and Gorwood, 2020).

Researchers and health professionals from Brazil, Norway and Portugal documented their health professional experiences facing this novel pandemic to advise health services. These countries were selected due to their position in the worldwide ranking on the 2019 Human Development (HD) Report (http://www.hdr.undp.org/en/2019-report). HD classifies countries according to indices such as economic and gender inequality, health, education, dignity and respect for human rights. Among the 100 countries in that report, Norway (1st), Portugal (40th) and Brazil (75th) are top, middle and bottom listed, respectively. Could the differences between these countries counteract the assistance to elderly in the COVID-19 pandemic age? This report aims to provide an overview of each country’s response from the authors’ perspective based on their informal interviews with a multidisciplinary health professional range.

## Brazil

To date, more than 30,000 confirmed COVID-19 cases and 2,000 deaths (fatality rate is approximately 6.3%) associated to the pandemic have been registered (Brasil, 2020). Deaths among the elderly correspond to 72% of all mortality cases (Brasil, 2020).

In Montes Claros, a city with more than 400,000 inhabitants, a team of researchers and health professionals aims to provide meals, hygiene products and social support to homeless, including the elderly. This initiative is called “Consultório na Rua (CR),” which has supported more than 98 older homeless (8% of the total city’s homeless population). Many challenges have emerged in the current COVID-19 epidemiological scenario. In face to deal with challenges, CR has provided greater attention to those persons. With civil society support, it has been possible to provide personal hygiene and safety products, such as soaps, toilet paper, toothpaste, toothbrushes, towels and fabric masks. All individuals received product cleaning and reuse guidance. In the first week of the influenza vaccine campaign, all the elderly assisted were vaccinated to minimize H1N1 flu syndrome. Inside the shelter, they were instructed to remain more isolated and to wear masks.

A psychology team from Hospital Aroldo Tourinho provided information about strategies used to manage older inpatients’ mental health with or without COVID-19 infection. They have been providing mental health support through videocalling via WhatsApp. Additionally, movies have been exhibited to minimize mental suffering.

The Neuroscience of Exercise Lab of Federal University of Rio de Janeiro (UFRJ) has offered elderly many physical activities to perform at home. They created some free-cost and public initiative videos to inform patients how to maintain physical activity and functional capacity during the quarantine and how to stay active. These videos were based on scientific evidence about exercise and mental health in older people and on the American College of Sports Medicine (ACSM) recommendations for the COVID-19 Pandemic and were shared via mobile phone, WhatsApp and social media, such as Facebook, Instagram and the website www.laboratoriolanex.com. These guidelines comprised several instructions about improving physical activity, how to do exercises to maintain strength, aerobic capacity and mobility, range of movement, balance and how to breathe correctly, meditate and relax. All resources were provided completely free of cost, as an important public initiative.

Old age psychiatry services are scarce in Brazil. To our knowledge, services provided by the UFRJ Institute of Psychiatry, the most specialized in Rio de Janeiro, are working partially. New admissions were canceled, while via telephone consultations are being provided just in urgent cases.

Finally, institutionalized older adults have received donations from private companies and from the general population to acquire hygiene and safety products. All group activities and external visits were suspended to avoid contamination. Little information about this population was available.

## Portugal

The epidemiological situation reported from the Directorate-General for Health (DGH), with data updated until 3 April 2020, indicated 10,524 confirmed cases of COVID-19 in Portugal with the following distribution among elderly population: 60–69 years (1,396 cases); 70–79 years (999); over 80 (1,242 cases). Of the 266 deaths recorded, 170 were over 80 years old, 60 were aged between 70 and 79, 24 between 60 and 69 (Moreira, 2020). Most probably, Portugal should face an increase of mental health problems in general, namely in vulnerable people.

Older adults’ institutionalization has demanded several strategies. A care-home director reported several actions that were implemented to manage COVID-19 crisis, relieve isolation and provide emotional support, such as organizing telephone calls, teleconference to connect residents with their families and engage family members in providing help. She has reported the difficulty to make instructions clear to the residents with cognitive impairment. She related, “it is difficult for them to understand instructions about social distancing or hand hygiene or restrictions on walking in the garden. So, it is important to be clear, use simple language and give special attention to this group.” Additionally, “some families have been facing the decision of keeping their loved ones at home or maintaining them in the care homes, and we must be aware that we have elderly aged between 80-90, and their offspring over 65, who sometimes present also mental health issues. Thus, in these circumstances elderly take care of elderly and this is emotionally complex.”

As soon as March 26, the Porto City Council Project was launched to implement a COVID-19 screening in the country, also for all elderly residing in care homes or collective residences at Porto, as well as people involved in institutions, reaching more than 1,500 people (Peixoto, 2020). ‘More recently, on April 6, Porto Municipality announced the extended screening to all hostels and shelters for the homeless, as well as residential homes that might welcome people with disabilities, something that should start after Easter (Porto, 2020a).

On March 31, Porto Municipality announced the transformation of the SuperBock Arena/Pavilhão Rosa Mota into the first special hospital created in Porto, with more than 300 beds and 27 wards, to combat the COVID-19 pandemic (Porto, 2020b). Also, the “Centro de Desporto da Universidade do Porto” offered online classes, specialized for seniors aged over 65, to provide simple physical exercises to perform at home in quarantine to maintain mobility and decrease isolation (Sampaio, 2020).

In Lisbon, a Solidarity Network was created to enable people who were not part of risk groups to register to help provide social support to the most vulnerable (Moreira, 2020). Over 1,200 people signed up, and they started to help the homeless, the isolated elderly or people identified as COVID-19 risk groups.

The voluntary campaign launched by the Government called “Care for All,” to support the elderly who lived in care homes during the pandemic, received more than 3,000 applications from people willing to help. The campaign aimed to strengthen the human resource teams in care homes and, thus, create rear networks for the employees of these institutions. About 15% of fatalities were elderly living in care homes (Silva, 2020).

Other informal support in the country has been provided by spontaneously organized solidarity networks: do grocery shopping, buy medicine and several local restaurants were continuing to cook meals for vulnerable people. Based on prior research (Ayalon, 2020), some countries data indicated a similar approach, an intergenerational solidarity. However, unfortunately ageism is also a worldwide reality. The pandemic is an opportunity to shift our thinking and call attention to these issues.

Portugal has reorganized and has adopted measures to tackle COVID-19 pandemic, and the Psychiatric services addressed these guidelines emitted by the DGH on 18 April 2020 (DGS, 2020).

## Norway

Norway has one of oldest populations worldwide. It is a public concern since the current pandemic has affected the elderly more than the younger population. Of 6,984 confirmed COVID-19 cases and 148 deaths (Norwegian Institute of Public Health, 2020), ‘1,599 people were infected abroad and 3,439 were infected locally within Norway.

All institutions that treated or performed elderly patients’ care have faced challenges and have taken actions. Both home-dwelling and nursing home residents were deeply affected. Many older persons living at home have become more isolated. At first, many have been afraid of being contaminated by COVID-19 and many have avoided going outside. Besides, all the activities have been suspended in this period, such as physical activities, social activities and physiotherapy. They have also avoided meeting family members, especially children and grandchildren, which increased the isolation. Homecare nurses have focused on good hand hygiene, maintaining social distance when possible and prioritizing visits. The unnecessary visits were suspended. Some measurements were taken to minimize the negative isolation consequences by promoting contact with family members by telephone or videocalls, performing active listening and talking about pleasurable themes when they were intensively worried (Goyal, 2020).

Most nursing home residents in Norway have a dementia diagnosis (more than 80%). Those with severe dementia might not understand the situation, but still, persons with dementia are more vulnerable to develop symptoms such as anxiety, depression and agitation. This can be triggered by situations they do not understand, like the daily routine changes powered by COVID-19 pandemic. Therefore, nursing homes in Norway have focused in maintaining daily routines, activities and especially measurements that stimulated pleasure. Many nursing homes have also offered contact with residents’ relatives through videocalls, and there has been positive feedback from both residents and relatives. Some relatives have also had contact with residents through glass walls or windows combined with telephone calls. A possible consequence of the suspension of routine physical activities and physiotherapy is the worsening of daily living function. Professionals have therefore tried to create not only other types of activities, physical activities but also social and cognitive stimulation. Another issue that might be frightening to the infected patients is to meet the professionals wearing infection control equipment. Consequently, this group needed to have contact with relatives through a screen or window.

Regarding old psychiatry wards, to minimize the infection risk, the non-critical psychiatry activities were canceled, such as outpatient clinic consultations and elective admissions. Some patients in need were followed up by telephone as often as they needed. For patients who needed to be admitted, there were rigid COVID-19 screening routines. Those admitted were neither allowed to have permission to go to cafeteria nor receive visitors, and the contact with relatives was limited to telephone calls or videocalls.

In conclusion, institutions with clinical contact with the elderly have faced the COVID-19 pandemic with procedures to minimize the risk of contamination and simultaneously protect the patients with care and treatment offer. Obviously, this has led to challenges such as shortage of health personal due to quarantine, less activity offer, no homecare visits, restrictions on consultations and admissions, suspension of family visits and minimization of educational activities. Negative consequences of the pandemic may arise, especially for patients that might have been neglected, for heavily burdened family members and staff and limited residents’ education as well as for the supervision of nursing homes. A study is planned in Norway to investigate the negative consequences of this long isolation.

## Final considerations

We noticed a common strategy to the three countries facing the pandemic: the use of technologies to bring the elderly closer to their relatives and friends and thus prevent them from feeling lonely, avoiding the risk of contamination and minimizing potential mental suffering. Health professionals from Brazil, Portugal and Norway used available technological devices to deliver mental health support. Flu vaccination campaign in Brazil and the screening of the vulnerable elderly, including homeless, in Portugal should be highlighted as great initiatives, since other countries with high HD index faced severe challenges to adopt these measures.

## Description of author(s)’ roles

RSMJ contacted Portuguese and Norwegian researchers, designed the manuscript, retrieved data from Brazil. LSFC retrieved data from Portugal and reviewed the manuscript critically. MLB and KMK retrieved data from Norway and reviewed the manuscript critically. CAS, DSH and LA retrieved data from Brazil. LLL drafted the work and reviewed it critically. ACD retrieved data from Brazil and reviewed the manuscript critically.
